# Clinical significance of SARS‐CoV‐2‐specific IgG detection with a rapid antibody kit for COVID‐19 patients

**DOI:** 10.1111/irv.12802

**Published:** 2020-09-10

**Authors:** Yong Chong, Hideyuki Ikematsu, Naoki Tani, Yoko Arimizu, Haruka Watanabe, Yukako Fukamachi, Akiko Yonekawa, Sho Iwasaka, Ruriko Nishida, Yoshihiro Eriguchi, Noriko Miyake, Shinji Shimoda, Yoji Nagasaki, Nobuyuki Shimono, Koichi Akashi

**Affiliations:** ^1^ Medicine and Biosystemic Science Kyushu University Graduate School of Medical Sciences (The First Department of Internal Medicine) Fukuoka Japan; ^2^ Japan Physicians Association Tokyo Japan; ^3^ Department of Infectious Disease National Hospital Organization Kyushu Medical Center Fukuoka Japan; ^4^ Emergency and Critical Care Center Kyushu University Hospital Fukuoka Japan; ^5^ Department of Clinical Chemistry and Laboratory Medicine Kyushu University Hospital Fukuoka Japan; ^6^ Center for the Study of Global Infection Kyushu University Hospital Fukuoka Japan

**Keywords:** a rapid kit, antibody, COVID‐19, IgG, PCR, SARS‐CoV‐2

## Abstract

**Background:**

The longitudinal observation of the detection of antibody responses to SARS‐CoV‐2 using antibody kits during the clinical course of COVID‐19 is not yet fully investigated.

**Objectives:**

To understand the significance of the detection of anti‐SARS‐CoV‐2 antibodies, particularly IgG, using a rapid antibody kit, during the clinical course of COVID‐19 patients with different severities.

**Methods:**

Sixty‐three serum samples from 18 patients (5 asymptomatic and 13 symptomatic patients) were retrospectively examined using a commercial SARS‐CoV‐2 IgM/IgG antibody kit. PCR positivity of patient samples was also examined as a marker of current SARS‐CoV‐2 infection.

**Results:**

IgG antibodies were detected in all cases in this study. The IgG detection rates reached 100.0% in samples collected on day 13 or later. IgG seropositivity after an initial negative status was observed in 13 patients (3/5 asymptomatic and 10/13 symptomatic cases). Interestingly, the persistence of both PCR and IgG positivity was detected in seven cases, of which three were asymptomatic. The longest overlap duration of the PCR and IgG positivity was 17 days in asymptomatic status.

**Conclusions:**

SARS‐CoV‐2‐specific IgG production can be detected in all infected individuals, using a rapid antibody kit, irrespective of clinical status. However, these findings suggest that, in some infected individuals, particularly those with asymptomatic status, the presence of virus‐specific IgG antibodies does not imply prompt viral clearance.

## 1 INTRODUCTION

The novel coronavirus disease (COVID‐19), caused by severe acute respiratory syndrome coronavirus 2 (SARS‐CoV‐2), has spread worldwide, and the World Health Organization (WHO) declared it a pandemic, on March 11, 2020. In Japan, the COVID‐19 outbreak has been accelerating since April 2020. A PCR assay of nasal and pharyngeal swab samples is generally used as the standard method for COVID‐19 diagnosis. On the other hand, serological testing based on the host's antibody responses to SARS‐CoV‐2 is being investigated to clarify how useful it is in the category of COVID‐19.^1^, ^2^ Rapid antibody detection kits, which can detect antibodies in human blood within a short time, are expected to be applied in the various situations of COVID‐19 management, and many, such kits are already used in the market. There is an ongoing verification of the accuracy of these antibody kits, in terms of sensitivity and specificity.^3^, ^4^ However, the longitudinal observation of the detection of antibody responses to SARS‐CoV‐2, using antibody kits, during the clinical course of COVID‐19, has not been fully investigated, although some studies have been reported.^5^, ^6^ We attempt to discuss the significance of the detection of anti‐SARS‐CoV‐2 antibodies, particularly IgG. In this study, we examined the detection of antibodies against SARS‐CoV‐2 and the relationship between seroconversion and PCR positivity, using an antibody kit in COVID‐19 patients with different degrees of severity. We also discuss the utility of a rapid antibody kit in the clinical and epidemiological setting of COVID‐19, based on the results of this study.

## 2 METHODS

### 2.1 Patients and sample collection

This retrospective study was conducted using samples obtained from patients admitted to the Kyushu University Hospital, Fukuoka, Japan. All patients had been diagnosed with COVID‐19 before admission, on the basis of a positive result from the real‐time PCR assay of nasal and pharyngeal swab specimens that was performed by the Japanese Institute of Health according to the manual for the detection of pathogen 2019‐nCoV.^7^ Serum samples, remaining from other biochemical tests, of 18 patients admitted between March and April 2020 were used for this study. During hospitalization, a PCR assay for COVID‐19 was performed by the Institute of Health. Clinical data were collected from electronic medical records. The Research Ethics Committee of Kyushu University Hospital approved this study.

### 2.2 Study definitions

The presence of fever was defined as an axillary temperature of 37.5°C or higher.^8^, ^9^ The severity of respiratory symptoms was graded as absent, mild, moderate, or severe.^9^ The patients were classified as symptomatic if they had at least a temperature higher than 37.5°C and/or moderate‐to‐severe respiratory symptoms.^9^ The symptomatic status was evaluated at the time of hospital admission. Asymptomatic patients were those who did not meet the symptomatic condition, neither before nor after admission. The severity of the symptomatic status was categorized as mild, severe, or critical. Mild symptomatic cases were characterized by blood oxygen saturation ≥93% and no oxygen requirement. Severe cases included blood oxygen saturation ≤92% and requirement of oxygen, delivered through a nasal cannula or an oxygen mask. Critical cases were those requiring mechanical ventilation for oxygen supply. For symptomatic patients, the day of symptoms onset (day 1) was considered as the beginning of the symptomatic status, as defined above. Day 1 for asymptomatic patients began on the first day of PCR positivity.

### 2.3 Antibody detection assay

An immunochromatographic assay kit, based on the recombinant nucleocapsid antigen of SARS‐CoV‐2 (2019‐nCoV IgG/IgM Rapid Test Cassette, Hangzhou Alltest Biotech Co. Ltd.), was used for rapid antibody detection, according to the manufacturer's instructions. In brief, 10 µl of serum was loaded into the sample port, followed by the addition of two drops (approximately 80 µl) of dilution buffer, to drive capillary action along the strip. The entire test took 10 minutes to complete. The presence of anti‐SARS‐CoV‐2 IgM and/or IgG antibodies was separately indicated by a red line in the corresponding area of the device.

## 3 RESULTS

A total of 63 samples from 18 patients were collected and analyzed in our study. Virus‐specific IgM antibodies were not detected in any of the samples, except for one (case 12), during the collection period ranging from day 2 to day 33 after symptoms onset, regardless of the number of days (1/63 samples, 1.6%) (Table 1).

**TABLE 1:** IgM detection sensitivity of the rapid immunochromatographic antibody kit

Case No.	Clinical status^a^	Days 1‐6^b^	Days 7‐9	Days 10‐12	Days 13‐
1	Asymptomatic	0/1	0/1	NA	0/1
2	Asymptomatic	NA	0/1	NA	0/1
3	Asymptomatic	0/1	NA	0/1	0/1
4	Asymptomatic	0/1	0/1	NA	NA
5	Asymptomatic	NA	0/1	NA	0/1
6	Symptomatic (critical)	NA	0/2	0/1	0/6
7	Symptomatic (critical)	NA	0/2	0/2	0/3
8	Symptomatic (severe)	NA	0/1	0/2	0/1
9	Symptomatic (severe)	NA	0/1	0/1	0/1
10	Symptomatic (severe)	0/2	NA	0/1	0/1
11	Symptomatic (mild)	NA	NA	NA	0/3
12	Symptomatic (mild)	NA	0/1	0/1	1/1
13	Symptomatic (mild)	NA	0/1	0/1	0/1
14	Symptomatic (mild)	NA	0/1	0/1	0/1
15	Symptomatic (mild)	NA	0/1	0/1	0/1
16	Symptomatic (mild)	0/1	0/1	0/2	NA
17	Symptomatic (mild)	0/1	0/1	0/1	NA
18	Symptomatic (mild)	0/1	0/1	NA	NA
Total		0/8 (0.0%)	0/17 (0.0%)	0/15 (0.0%)	1/23 (4.3%)

Abbreviation: NA, not available.

^a^ Clinical status is defined in the section of Methods.

^b^ Days indicate the day after symptoms onset.

Virus‐specific IgG antibodies were detected in all cases during the collection period (18/18 cases, 100.0%) (Table 2). IgG antibodies were not detected in samples collected by day 6 after symptoms onset (0/8 samples, 0.0%). The detection rate of IgG was 41.4% (7/17 samples) in samples collected from day 7 to 9 after symptoms onset. Of these, four samples were from asymptomatic patients. The IgG detection rates increased to approximately 70% during days 10‐12 after symptoms onset and reached 100.0% in samples collected on day 13 or later after symptoms onset.

**TABLE 2:** IgG detection sensitivity of the rapid immunochromatographic antibody kit

Case No.	Clinical status^a^	Days 1‐6^b^	Days 7‐9	Days 10‐12	Days 13‐
1	Asymptomatic	0/1	1/1	NA	1/1
2	Asymptomatic	NA	1/1	NA	1/1
3	Asymptomatic	0/1	NA	0/1	1/1
4	Asymptomatic	0/1	1/1	NA	NA
5	Asymptomatic	NA	1/1	NA	1/1
6	Symptomatic (critical)	NA	0/2	1/1	6/6
7	Symptomatic (critical)	NA	0/2	1/2	3/3
8	Symptomatic (severe)	NA	0/1	1/2	1/1
9	Symptomatic (severe)	NA	1/1	1/1	1/1
10	Symptomatic (severe)	0/2	NA	1/1	1/1
11	Symptomatic (mild)	NA	NA	NA	3/3
12	Symptomatic (mild)	NA	0/1	1/1	1/1
13	symptomatic(mild)	NA	0/1	0/1	1/1
14	Symptomatic (mild)	NA	1/1	1/1	1/1
15	Symptomatic (mild)	NA	0/1	1/1	1/1
16	Symptomatic (mild)	0/1	0/1	1/2	NA
17	Symptomatic (mild)	0/1	0/1	1/1	NA
18	Symptomatic (mild)	0/1	1/1	NA	NA
Total		0/8 (0.0%)	7/17 (41.1%)	10/15 (66.7%)	23/23 (100.0%)

Abbreviation: NA, not available.

^a^ Clinical status is defined in the section of Methods.

^b^ Days indicate the day after symptoms onset.

**FIGURE 1:**
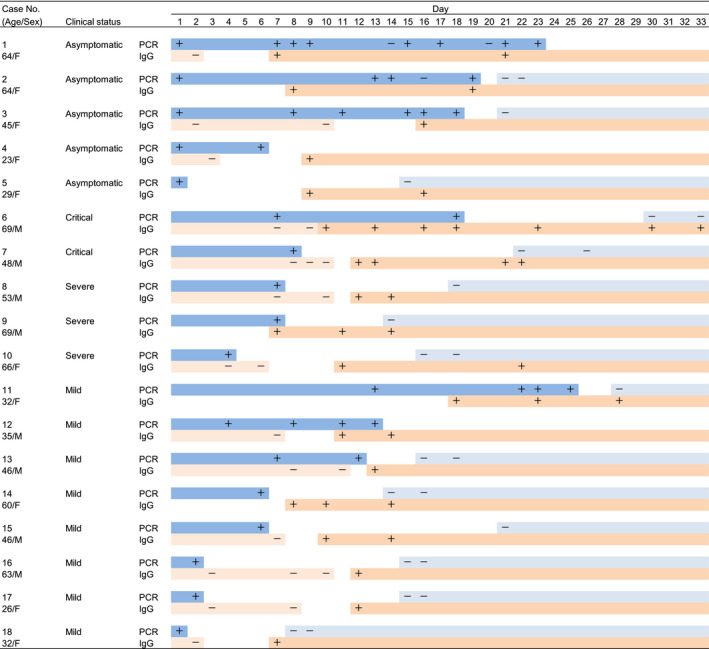
Clinical course of anti‐SARS‐CoV‐2 IgG and PCR positivity. The collection period of this study was from day 1 to day 33. PCR+/− in each case indicates positive and negative PCR results, respectively, on the day when PCR testing was performed after day 1. IgG+/− in each case indicates positive and negative IgG results, respectively, on the day when antibody kit testing was performed after day 1. The dark blue bars indicate the duration between day 1 of symptoms onset and the last day of a positive PCR result. The light blue bars indicate the duration between the first day of a negative PCR result after the last day of a positive PCR result and day 33. The light orange bars indicate the duration between day 1 of symptoms onset and the last day of a negative IgG result. The dark orange bars indicate the duration between the first day of a positive IgG result and day 33. The outlines indicate the duration that is not evident for PCR or IgG positivity in each case. Clinical status is defined in the Methods section. Day indicates the day after symptoms onset. Day 1 for asymptomatic patients began on the first day of PCR positivity

Figure 1 shows the clinical course of each case, with the respective PCR and IgG positivity. The last day on which a negative IgG result was observed was day 11 in case 13. A total of 13 patients (3/5 asymptomatic, 4/5 critical and severe, and 6/8 mild cases) became seropositive for IgG antibodies after an initial negative status. In seven of the 13 patients, the maintenance of IgG positivity after seroconversion was observed, and in this study, no patient was found to become seronegative after seroconversion, even up to day 33 (case 6). These findings suggested that anti‐SARS‐CoV‐2 IgG antibodies could be produced regardless of the symptomatic status or severity of the disease. A negative PCR result prior to the first day of a positive IgG result was not detected in any cases. Both PCR and IgG positivity on the same collection day were detected in seven cases (cases 1, 2, 3, 6, 9, 11, and 12), of which three were asymptomatic (day 21 in case 1, day 19 in case 2, and day 16 in case 3). Assuming a positive IgG result persists after the first positive day during the collection period in this study, we can estimate the overlap duration of both PCR and IgG positivity. In the seven cases, the overlap duration was 17 days in case 1, 12 days in case 2, 3 days in case 3, 9 days in case 6, 1 day in case 9, 8 days in case 11, and 3 days in case 12. The longest overlap duration was 17 days in an asymptomatic case (case 1).

## 4 DISCUSSION

The production of anti‐SARS‐CoV‐2 IgG antibodies was observed in all COVID‐19 patients approximately 10 days after symptoms onset, irrespective of symptom status, or disease severity. There is a general assumption that virus‐specific IgG production elicited by class switch can result in prompt viral elimination; however, the duration of PCR positivity after the detection of anti‐SARS‐CoV‐2 IgG was not similar in the COVID‐19 patients examined in this study. Thus, in some patients, both PCR and IgG positivity persisted in their clinical course. Prolonged viral shedding after antibody seroconversion was shown as a case report.^10^ Recently, Lee et al reported that symptomatic patients with development of anti‐SARS‐CoV‐2 IgM antibodies had a shorter duration of a positive PCR result than those without the presence of anti‐SARS‐CoV‐2 IgM antibodies, and their data implied that a prolonged PCR positivity was observed in asymptomatic patients rather than in symptomatic ones.^5^

Therefore, it is intriguing that the prolonged overlap duration of PCR and IgG positivity was observed in asymptomatic patients in this study. This is a new finding that has not been indicated yet, to our best knowledge. In patients with few symptoms, appropriate immune responses might not have occurred, possibly due to the differential quantity or quality of the immune responses. The detection of antibodies to the nucleocapsid protein of SARS‐CoV‐2 was examined in this study. It may be attractive to investigate antibodies to other proteins of the virus, particularly the spike protein, which is the main antigen that elicits neutralizing antibodies. We need to further investigate the immunological mechanisms by which the delay of viral elimination occurs, even after the production of SARS‐CoV‐2‐specific IgG antibodies.

The detection rate for anti‐SARS‐CoV‐2 IgM antibodies in the kit in this study was extremely low. Virus‐specific IgM antibodies are generally produced faster than IgG antibodies after viral exposure. Other assays for anti‐SARS‐CoV‐2 IgM and IgG antibodies reported that it was difficult to selectively detect virus‐specific IgM before IgG within 7 days after symptoms onset.^11^, ^12^, ^13^ Other rapid antibody kits also showed an IgM sensitivity of ~10% during an early infection stage.^3^ Detection of anti‐SARS‐CoV‐2 IgM before IgG might be difficult due to the low detection sensitivity, regardless of the specific assay. Currently, serological testing, including rapid antibody kits, is not useful for the diagnosis of COVID‐19 in the setting of acute illness.

In contrast, the detection rate of anti‐SARS‐CoV‐2 IgG antibodies using the kit used in this study reached 100.0% 2 weeks after symptoms onset. Similarly, other rapid antibody kits showed a sensitivity of ~100.0% for IgG antibodies 2 weeks after symptoms onset.^3^, ^14^ Addressing the precise specificity of the kit used in this study for the detection of anti‐SARS‐CoV‐2 IgG is difficult due to the lack of non‐infected samples. It was originally reported that the specificity was 98.0% for the performance of this kit.^15^ The false positivity for anti‐SARS‐CoV‐2 IgG with this kit has been addressed, and no false‐positive results have been found among more than 100 previously collected serum samples (Ikematsu et al manuscript in submission). Therefore, the specificity for anti‐SARS‐CoV‐2 IgG of the kit used in this study is presumed to be extremely high.

This study indicated that the kit was able to identify cases where infection had resolved after recovery from COVID‐19. In addition, asymptomatic patients with negative PCR and positive IgG results were included. Other antibody assays (not rapid kits) showed that asymptomatic close contacts of COVID‐19 patients were PCR‐negative and IgG‐positive.^12^, ^16^ Rapid and simple antibody kits could represent useful and powerful tools for tracing close contacts of COVID‐19 patients, identifying previously infected healthcare workers, and surveying subclinical infected individuals in the community.

In this study, patients with positive PCR and IgG results were detected during hospitalization. Patients whose results are positive after PCR assays of nasal and pharyngeal swab specimens are clinically regarded as currently infected with SARS‐CoV‐2. Positive results of both PCR and IgG suggested that individuals with anti‐SARS‐CoV‐2 IgG antibodies might include not only previously infected cases, but also currently infected ones. In particular, the detection of positive PCR and IgG results in asymptomatic patients is significant. Assuming that these patients are in the community, without hospitalization, and undergo rapid antibody kit testing, their IgG positivity could suggest a current SARS‐CoV‐2 infection. Thus, asymptomatic individuals with anti‐SARS‐CoV‐2 IgG antibodies could be “silent spreaders” in this pandemic situation.

This study had some limitations. First, the sample size was small. Although the findings obtained from the asymptomatic patients were interesting, only five asymptomatic subjects were enrolled. Second, the study was retrospective, and therefore, it was not possible to evaluate the precise timing of IgG seroconversion or PCR negativity because of the lack of a prospective sample collection. Third, the presence of anti‐SARS‐CoV‐2 antibodies detected by the kit used in the study should be verified through quantification assays. Nevertheless, the findings of this study are informative. They indeed suggest that the detection of anti‐SARS‐CoV‐2 IgG antibodies can be observed in all infected individuals, regardless of clinical status that viral clearance after IgG production is not similar and that a delay of viral elimination, even after IgG production, can be observed, particularly in some individuals with asymptomatic status.

## CONFLICT OF INTEREST

We have no financial conflicts of interest to declare.

## AUTHOR CONTRIBUTION

**Yong Chong:** Conceptualization (lead); Data curation (lead); Formal analysis (lead); Investigation (lead); Project administration (lead); Writing‐original draft (lead); Writing‐review & editing (lead). **Hideyuki Ikematsu:** Conceptualization (equal); Writing‐original draft (supporting). **Naoki Tani:** Data curation (equal). **Yoko Arimizu:** Data curation (supporting). **Haruka Watanabe:** Data curation (supporting). **Yukako Fukamachi:** Data curation (supporting). **Akiko Yonekawa:** Data curation (supporting). **Sho Iwasaka:** Data curation (supporting). **Ruriko Nishida:** Data curation (supporting). **Yoshihiro Eriguchi:** Data curation (supporting). **Noriko Miyake:** Data curation (supporting). **Shinji Shimoda:** Project administration (supporting). **Yoji Nagasaki:** Data curation (supporting). **Nobuyuki Shimono:** Project administration (supporting). **Koichi Akashi:** Project administration (supporting).
